# Effects of Selenium Nanoparticle Application on Flavor Volatiles of Aromatic Rice

**DOI:** 10.3390/foods14040552

**Published:** 2025-02-07

**Authors:** Haowen Luo, Simin Zhang, Xiaojuan Pu, Longfei Xia, Wentao Yi, Xianghai Yu, Changjian Zuo, Xiangru Tang

**Affiliations:** 1 State Key Laboratory for Conservation and Utilization of Subtropical Agricultural Bioresources, College of Agriculture, South China Agricultural University, Guangzhou 510642, China; luohaowen@stu.scau.edu.cn (H.L.); 15989204044@163.com (X.P.); xialongfei@stu.scau.edu.cn (L.X.); ywt1999@stu.scau.edu.cn (W.Y.); 2 Scientific Observing and Experimental Station of Crop Cultivation in South China, Ministry of Agriculture, Guangzhou 510642, China; 3 Guangzhou Key Laboratory for Science and Technology of Aromatic Rice, Guangzhou 510642, China; 4 College of Agriculture, South China Agricultural University, Guangzhou 510642, China; zsm446972239@163.com; 5 Green Huinong Biotechnology (Shenzhen) Co., Ltd., Shenzhen 518107, China; 13352928378@163.com (X.Y.); 34333824@163.com (C.Z.)

**Keywords:** aroma, cropping season, flavor, fragrant rice, nanosized materials, selenium, volatile

## Abstract

Aromatic rice is famous for its pleasant aroma which consists of many flavor volatiles. The present study was to explore the effects of selenium nanoparticle (SeNP) application on flavor volatiles of aromatic rice based on a worldwide database of flavor molecules accessed on November 19 2024. A field experiment was carried out with the foliar application of SeNP at early growth stage (S1), middle growth stage (S2), and late growth stage (S3) of aromatic rice plants in two cropping seasons. In the control group (CK), no selenium-based treatment was applied. There were in total 27 and 24 flavor volatiles registered in FlavorDB2 detected in aromatic rice in the early and late cropping seasons, respectively. The flavors that appear most often were fat, fresh, fruit, aldehydic, green, sweet, citrus, and waxy. Compared with CK, S3 treatment caused the absence of 5 and 4 flavor volatiles in the early and late seasons, respectively. S2 treatment caused the exclusive presence of 2-undecenal and 3-hexenal,(Z)- in the early season and the exclusive presence of 2-hexenoic acid and decanal in the late season. The results of principal components analysis (PCA) showed that S2 and S3 treatments substantially impacted the flavor volatiles of aromatic rice in the early season while S1 and S2 treatments substantially impacted the flavor volatiles of aromatic rice in the late season. There were 12 and 4 differential flavor volatiles found in the early and late cropping seasons respectively. S2 treatment significantly increased the content of 10 flavor volatiles including 2-acetyl-1-pyrroline, benzaldehyde, 2-hexenoic acid, hexanoic acid, octanal, 2-octenal,(E)-, heptanal, 2,4-heptadienal,(E,E)-, 3-hexenoic acid,(E)-, and n-hexadecanoic acid. In addition, the effects of SeNP on flavor volatiles varied between different cropping seasons indicated that climate had a substantial impact on flavor volatiles in aromatic rice. Overall consideration, the heading stage, i.e., the middle growth stage, is the most suitable stage to apply SeNP to maximize the benefits on the flavor volatiles of aromatic rice.

## 1. Introduction

Rice (*Oryza sativa* L.) is a food crop in China and many other Asia countries and feeds more than half the population in the world. In recent years, more attention has been drawn to rice quality because of the rapid development of society and the continuous improvement of people’s living standards. The demand for rice with better quality is rising despite it has a higher price in the market. The price of rice mainly depends on the rice quality attributes which includes nutrients, appearance, taste, and aroma. As one of the important factors determining rice quality, aroma is the combined effect of numerous flavor volatiles. Aromatic rice is a special rice known for its pleasant aroma which contains a lot of volatile compounds. An earlier study [1] divided these volatiles into several classes, including fatty acid derivatives, terpenoids, phenylpropanoids (benzenoids), and amino acid derivatives. A previous research detected more than 400 volatile compounds in aromatic rice through a GC × GC-TOF-MS determination and analysis [2]. Recently, Li et al. [3] indicated that 2-acetyl-1-pyrroline, trans-2-octenal, hexanal, octanal, 1-octen-3-ol, 2-pentylfuran, decanal, trans-2nonenal, and trans, trans-2,4-decadienal were the potential characteristic volatiles in rice aroma.

During the field production of aromatic rice, agronomic practices such as water management, plant growth regulators, and cultivation modes could remarkably impact the flavor volatiles of aromatic rice, especially 2-acetyl-1-pyrroline. Many studies have revealed that the input of fertilizer, water management, and climate conditions during the cultivation of aromatic rice plants significantly affected the formation of 2-acetyl-1-pyrroline [4,5,6]. Our previous study also found that the application of the organic cultivation pattern not only increased 2-acetyl-1-pyrroline content but also caused the absence of 56 volatiles and the exclusive presence of 10 new volatiles compared to the traditional cultivation pattern [2]. However, most studies about the regulation in aromatic rice aroma focused on 2-acetyl-1-pyrroline, while other volatiles were rarely reported. Therefore, it is important to suitably adjust agronomic practices in aromatic rice production for the goal of achieving better rice aroma. 

The input of selenium (Se) fertilizer in crop cultivation could greatly increase the yield and improve the quality [7,8]. The improvements in crop productivity and quality due to Se are attributed to its ability to substantially enhance the photosynthesis and antioxidants of crops [9,10]. Furthermore, a previous study indicated that Se fertilizer management could promote nitrogen metabolism and nitrogen use efficiency in wheat plants [11]. Selenium nanoparticle (SeNP) is a new kind of Se fertilizer that offers superior stability, high bioavailability, excellent particle dispersion, and strong antioxidant capabilities, with minimal toxicity concerns compared with traditional Se fertilizers [12,13]. An earlier study showed a higher grain yield of rice plants under the foliar application of SeNP than both the applications of organic Se and inorganic Se [14]. The study by Huang et al. [15] also found that foliar application of SeNP increased yield, soluble solid content, soluble sugar, reducing sugar, and dry matter of radish. However, the effects of SeNP on flavor volatiles of aromatic rice have not been reported. 

Food aroma is exhibited by the interaction of molecules via gustatory and olfactory mechanisms and understanding the pivotal role of flavor molecules in food and fragrances, the existence of a comprehensive repository is important for the related research. In recent years, a worldwide database of flavor molecules (https://cosylab.iiitd.edu.in/flavordb2/ (accessed on 26 November 2024), FlavorDB2) with continuous updates has been established to help the development of scientific research for the flavor and fragrance community [16]. Therefore, we carried out a field experiment with two cropping seasons and three SeNP applications at different growing stages of aromatic rice. The volatiles were determined and analyzed through a GCMS-QP approach. The objective of the current study was to primarily explore the impacts of SeNP application on the flavor volatiles of aromatic rice based on FlavorDB2. Our study will provide new formations for rice aroma and aromatic rice cultivation.

## 2. Materials and Methods

### 2.1. Plant Materials and Field Conditions

A field experiment was carried out in the Experimental farm of South China Agricultural University, Guangzhou City, Guangdong Province, China (23°14′ N, 113°38′ E), in 2023. The climatic type of this trial site is a subtropical monsoon climate. An aromatic rice cultivar, “19Xiang”, was used as the plant material. The experimental soil was sandy loam with 17.55 g kg^−1^ organic matter, 1.03 g kg^−1^ total nitrogen, and 5.80 pH. The crop management was followed by our previous research [17] and the meteorological data during the field experiment are shown in Table 1. The early cropping season is between April and June (transplanting in early April and harvest in late June). The late cropping season is between April and June (transplanting in early August and harvest in late October).

### 2.2. Experimental Design

The experiment was arranged in a randomized complete block design (RCBD) in triplicate with a net plot size of 12.5 m^2^. Four treatments were adopted as (CK) no Se application, (S1) foliar application of SeNP at the early growth stage (the end of the tillering stage), (S2) foliar application of SeNP at the middle growth stage (the heading stage), and (S3) foliar application of SeNP at the late growth stage (the grain-filling stage) of aromatic rice plants. The applied Se concentration was 6.67 mg L^−1^ in each plot. The choice of the applied stages and concentration was based on the previous works from our team [17] and others [14,15]. The SeNP used in the present study was a simple substance Se produced by Green Huinong Biotechnology (Shenzhen) Co., Ltd., China.

### 2.3. Plant Sampling and Determination of Flavor Volatiles

**Figure 1:**
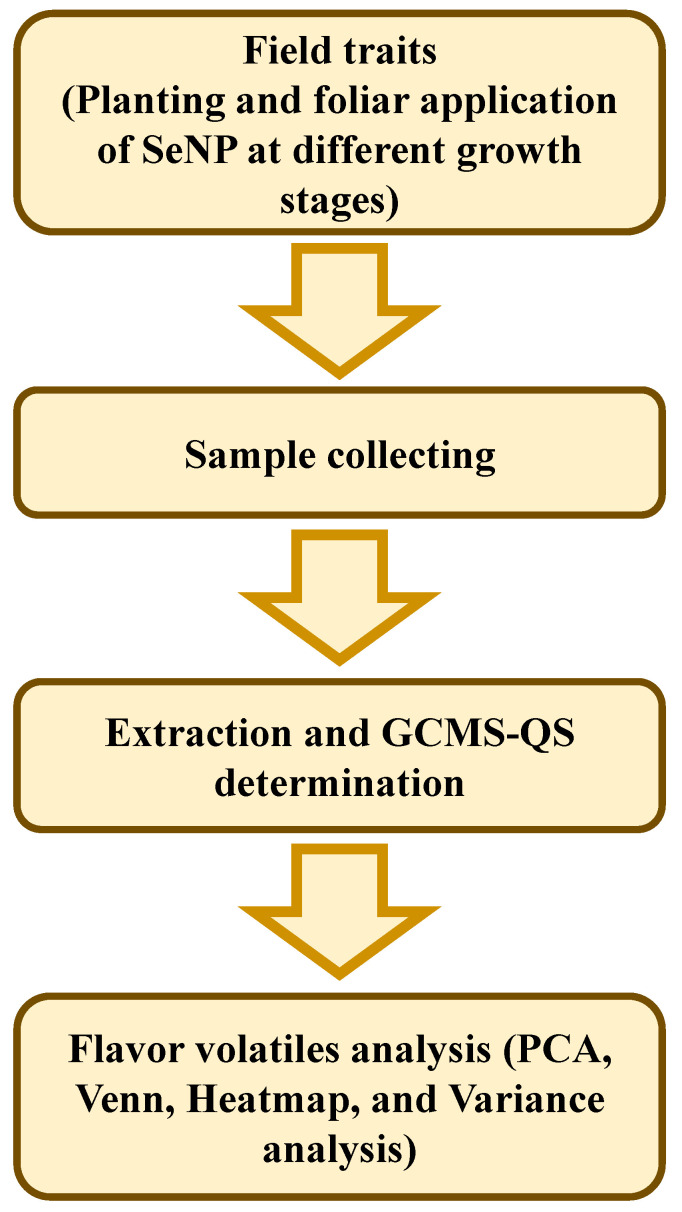
The flowchart from a field experiment to volatile analysis in the present study.

At the harvest, the mature grains were collected in each plot and stored at −80 °C until the determination of volatiles. The determination was carried out according to our previous study [18]. The grain sample was ground to powder with liquid nitrogen. The samples were added to dichloromethane. After the extraction, the supernatant was transferred to GCMS-QP 2010 Plus (Shimadzu Corporation, Kyoto city, Japan) for analysis. A flowchart explaining the treatments and analysis steps is shown in Figure 1. 

### 2.4. Statistical Analysis

Experimental data in the present study were analyzed using statistical software ‘Statistix8.1′ (Analytical Software, Tallahassee, FL, USA) while differences among means were separated using the least significant difference (LSD) test at the 5% probability level. The principal components analysis (PCA), Venn plot, heatmap, and association diagram between flavor volatiles and flavors were conducted on the BioDeep Platform (https://www.biodeep.cn) accessed on 19 November 2024. The figures were made and represented using Sigma Plot 14.0 (Systat Software Inc., San Jose, CA, USA).

## 3. Results and Discussion

### 3.1. The Number and Type of Flavor Volatiles After SeNP Application

**Figure 2:**
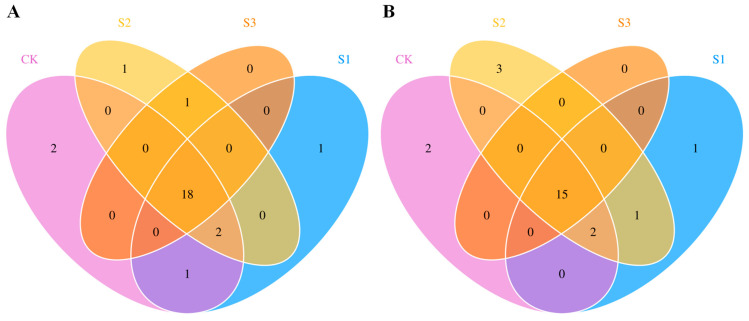
Venn plot for flavor volatiles in aromatic rice under different SeNP treatments. (**A**) for early cropping season. (**B**) for late cropping season.

There were in total 27 and 24 flavor volatiles registered in FlavorDB2 detected in aromatic rice in the early and late cropping seasons, respectively (Table 2). Compared with CK, SeNP treatments not only caused the exclusive presence of some flavor volatiles but also the absent of some flavor volatiles. In the early cropping season (Figure 2A), compared with CK, S1 treatment caused the exclusive presence of D-limonene which has been reported in the study of Luo et al. [19] who demonstrated that foliar application of phenylalanine significantly increased D-limonene content in aromatic rice. As a flavor volatile with flavors of mint, lemon, citrus, orange, fresh, and sweet, D-limonene is a monocyclic monoterpene that possesses potent therapeutic effects [20]. However, this compound occurs in all the samples in the late seasons which means that the climate conditions might have a great impact on D-limonene biosynthesis in aromatic rice. Meanwhile, we observed that all SeNP treatments caused the absence of tetradecane and 2-decenal,(E)- in the early season. The study by Hinge et al. [1] also found tetradecane in aromatic rice and the study by Gokila et al. [21] indicated that tetradecane might be one of the main herbivore-induced volatiles which play an important part in attracting insect pests and other natural enemies. But tetradecane was not detected in the late cropping season and thus, we deduced that foliar application of SeNP might help to control the pest in the early season by causing the absence of this herbivore-induced volatile. Moreover, we found that S2 and S3 treatments caused the exclusive presence of 2-undecenal in the early cropping season. In the late season (Figure 2B), S1 treatment caused the exclusive presence of 2-butanone with flavors of ethereal, ether, fruity, acetone, and camphor whilst this 2-butanone was not detected in the early season. Previous studies indicated that 2-butanone mainly exists in the rice bran layer and its content or existence varied largely from aromatic rice varieties [22,23]. In addition, we observed that the least number of flavor volatiles was recorded in S3 treatment in both cropping seasons, and compared with CK, S3 treatment caused the absence of 5 and 4 flavor volatiles in early and late seasons, respectively.

### 3.2. Relation Between Flavor Volatiles and Flavors

**Figure 3:**
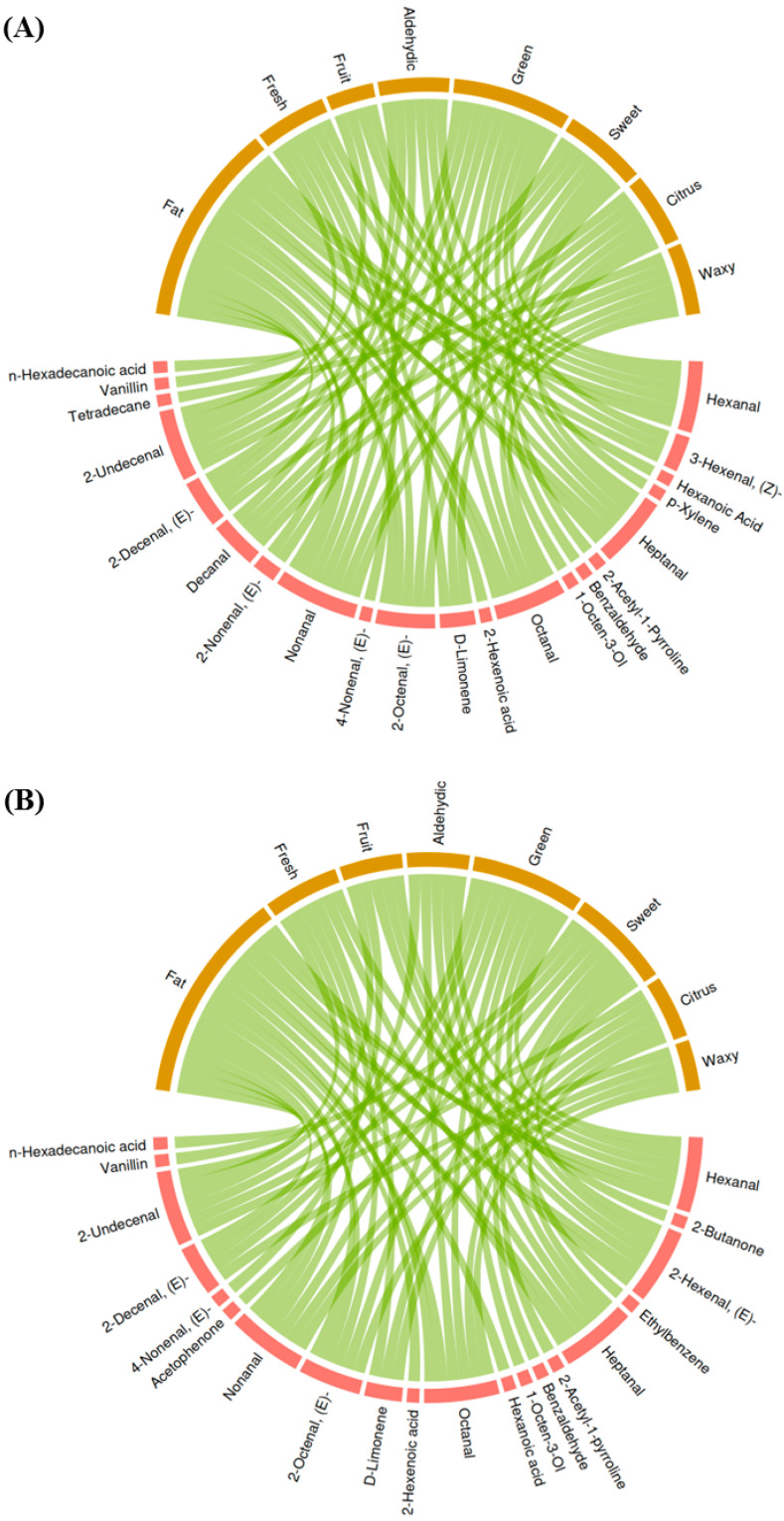
The association diagram between flavor volatiles and flavors. (**A**) for the early season. (**B**) for the late season.

According to Table 2, the flavors that appear most often were fat, fresh, fruit, aldehydic, green, sweet, citrus, and waxy. After the classification process, we constructed an association diagram (Figure 3) and found that these 8 flavors were connected to 21 and 20 flavor volatiles in early and late seasons, respectively. Interestingly, we observed that volatiles such as 2-undecenal, 2-decenal,(E)-, decanal, nonanal, octanal, heptanal, and hexanal contributed more than one of these flavors. Among these volatiles, heptanal, hexanal, nonanal, octanal, and decanal have been reported to be closely related to the sensory flavors of aromatic rice [3]. An earlier study indicated that these volatiles might be important contributors to the flavor of rice [3]. However, there are a lack of public standards on how to calculate the precise contribution of these flavor volatiles with their content to the rice flavors, and thus, it is difficult to determine the effects of treatments on rice flavors.

### 3.3. PCA of Flavor Volatiles

**Figure 4:**
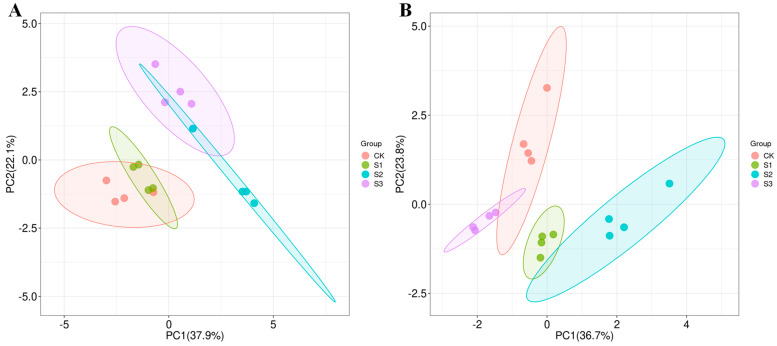
PCA score plot of flavor volatiles in aromatic rice under different SeNP applications. (**A**) for the early season. (**B**) for the late season.

**Figure 5:**
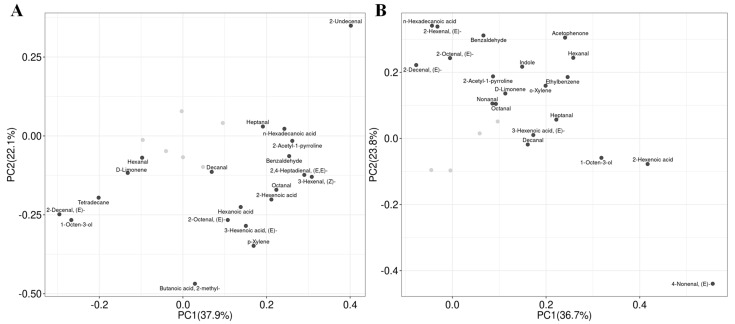
PCA loading plot of flavor volatiles in aromatic rice under different SeNP applications. (**A**) for the early season. (**B**) for the late season.

PCA is a commonly used data dimensionality reduction technique and an important statistical analysis method. According to the PCA results (Figure 4),in the early season, the first axis separated S2 from CK while S3 was separated from CK by both the second axes. In the late season, the first axis separated S2 from CK while the second separated S1 and S3 from CK although CK and S3 were closely grouped. The score plot revealed that S2 and S3 treatments substantially impacted the flavor volatiles of aromatic rice in the early season while S1 and S2 treatments substantially impacted the flavor volatiles of aromatic rice in the late season. Our results agreed with the study by Ruan et al. [2], which showed that volatiles of aromatic rice could be impacted by agronomic practices. The PCA loading plot (Figure 5) showed that in the early season, S2 treatment was distinguished from CK by the higher content of heptanal, n-hexadecanoic acid, 2-acetyl-1-pyrroline, benzaldehyde, decanal, 2,4-heptadienal,(E,E)-, 3-hexenal,(Z)-, octanal, 2-hexenoic acid, hexanoic acid, 2-octenal,(E)-, 3-hexenoic acid,(E)-, p-xylene, butanoic acid,2-methyl- and lower content of hexanal, D-limonene, tetradecane, 2-decenal,(E)-, and 1-octen-3-ol. Meanwhile, S3 treatment was distinguished from CK by the higher content of heptanal, n-hexadecanoic acid, and the exclusive presence of 2-undecenal. In the late season, S1 treatment was distinguished from CK by the higher content of 1-octen-3-ol, 2-hexenoic acid, and the lower content of n-hexadecanoic acid, 2-hexenal,(E)-, benzaldehyde, acetophenone, 2-octenal,(E)-, indole, hexanal, 2-acetyl-1-pyrroline, D-limonene, ethylbenzene, o-xylene, nonanal, octanal, and heptanal. Meanwhile, S2 treatment was distinguished from CK by the higher content of benzaldehyde, acetophenone, hexanal, indole, 2-acetyl-1-pyrroline, ethylbenzene, D-limonene, o-xylene, nonanal, octanal, heptanal, 3-hexenoic acid,(E)-, decanal, 1-octen-3-ol, 2-hexenoic acid, and lower content of n-hexadecanoic acid, 2-hexenal,(E)-, and 2-decenal,(E)-. Among these volatiles, D-limonene, 1-octen-3-ol, 2-acetyl-1-pyrroline, o-xylene, and octanal were previously reported in the research on aromatic rice aroma while 2-acetyl-1-pyrroline was considered as the main volatile that impacts the aroma under different cultivation modes [3,4,5,6,19].

### 3.4. Effects of Nano-Se on Contents of Different Flavor Volatiles

**Figure 6:**
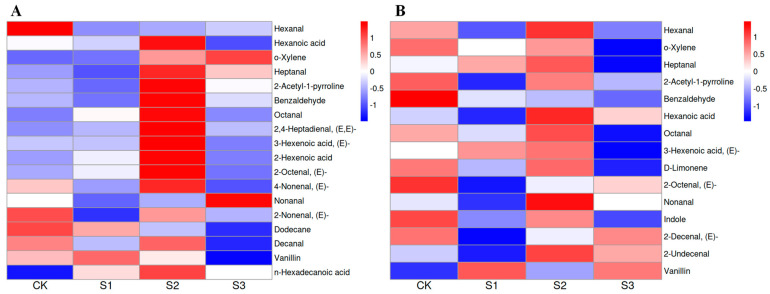
Heatmap of the common flavor volatiles among all treatments. (**A**) for the early season. (**B**) for the late season.

**Figure 7:**
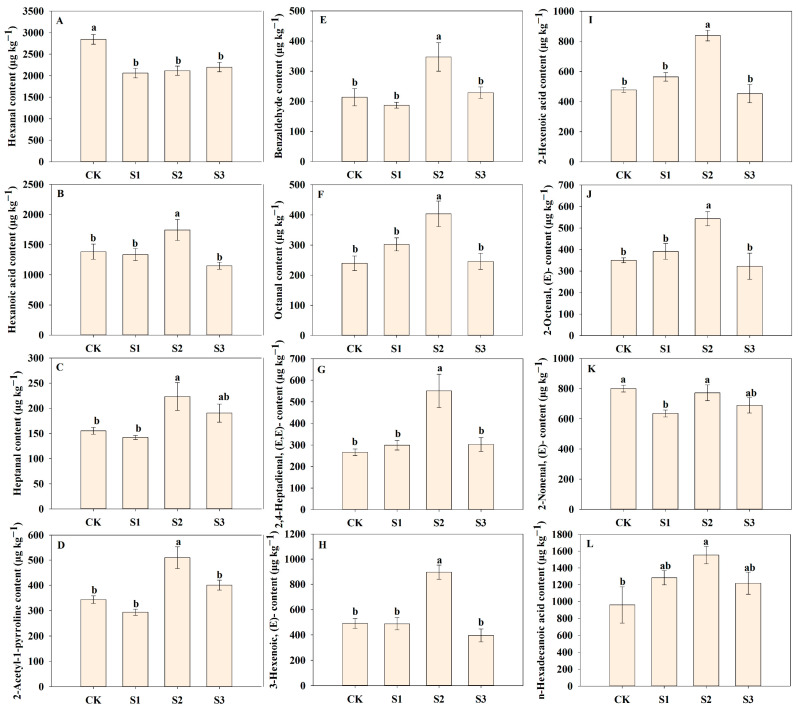
The differential flavor volatiles in the early seasons. Different letters represent significant differences between treatments with *p*-values < 0.05. (**A**) for Hexanal; (**B**) for Hexanoic acid; (**C**) for Heptanal; (**D**) for 2-Acetyl-1-pyrroline; (**E**) for Benzaldehyde; (**F**) for Octanal; (**G**) for 2,4-Heptadienal, (E,E)-; (**H**) for 3-Hexenoic acid, (E)-; (**I**) for 2-Hexenoic acid; (**J**) for 2-Octenal, (E)-; (**K**) for 2-Nonenal, (E)-; (**L**) for n-Hexadecanoic acid.

**Figure 8:**
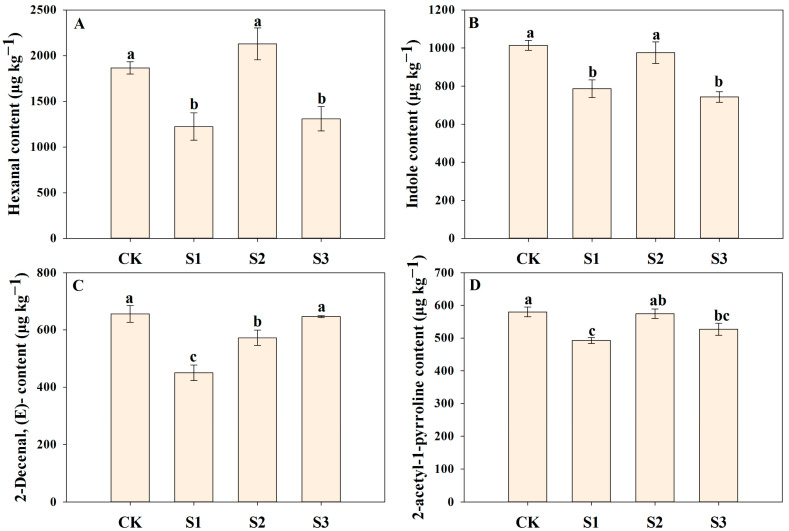
The differential flavor volatiles in the late seasons. Different letters represent significant differences between treatments with *p*-values < 0.05. (**A**) for Hexanal; (**B**) for Indole; (**C**) for 2-Decenal, (E)-; (**D**) for 2-Acetyl-1-pyrroline.

Foliar application of SeNP impacts the contents of some common flavor volatiles among all treatments (Figure 6, Figure 7 and Figure 8). There were 12 and 4 differential flavor volatiles found in the early and late cropping seasons, respectively. In the early season, compared with CK, S2 treatment significantly increased 2-acetyl-1-pyrroline content by 48.65% in the early season, which agreed with our previous study [17]. 2-Acetyl-1-pyrroline is considered a key flavor volatile in aromatic rice aroma with flavors of roast, nut, roasted, ham, sweet, nutty [24]. Luo et al. [25] indicated that Se enhanced 2-acetyl-1-pyrroline biosynthesis in aromatic rice by regulating the transcript levels of related genes as well as the contents of related precursors. However, we observed that the effects of SeNP on 2-acetyl-1-pyrroline content varied from the cropping seasons and the applied stages. It might be explained by the great effects of climate conditions and genetic factors considering 2-acetyl-1-pyrroline content varies largely from varieties [26] and is sensitive to light and temperature [5,27]. Furthermore, a similar phenomenon was observed on some other flavor volatiles such as o-xylene, heptanal, and vanillin in aromatic rice under SeNP treatments in different cropping seasons, which indicated that climate might have a great impact on the formation of more flavor volatiles in aromatic rice plants. Besides 2-acetyl-1-pyrroline, S2 treatment remarkably improved the content of benzaldehyde, 2-hexenoic acid, hexanoic acid, octanal, 2-octenal, (E)-, heptanal, 2,4-heptadienal,(E,E)-, 3-hexenoic acid, (E)-, and n-hexadecanoic acid by 62.70%, 75.49%, 26.07%, 68.40%, 55.24%, 43.69%, 106.94%, 82.50%, and 61.91%, respectively compared with CK in the early cropping season. Among these flavor volatiles, 2,4-heptadienal,(E,E)-, 2-octenal,(E)-, and octanal were previously reported as the potential key contributors to rice aroma [28]. In addition, although there were differences in the effects of SeNP application between two cropping seasons, S2 treatment exhibited the most up-regulated flavor volatiles than other SeNP treatments, and thus, we recommended the heading stage, i.e., the middle growth stage, the most suitable stage to apply SeNP to maximize the benefits on the flavor volatiles of aromatic rice.

## 4. Conclusions

Foliar application of SeNP substantially impacted the flavor volatiles of aromatic rice in different cropping seasons. In the present study, 27 and 24 flavor volatiles registered in FlavorDB2 detected in aromatic rice were detected in the early and late cropping seasons, respectively. The flavors that appear most often were fat, fresh, fruit, aldehydic, green, sweet, citrus, and waxy. Compared with CK, S3 treatment caused the absence of 5 and 4 flavor volatiles in the early and late seasons, respectively. S2 treatment caused the exclusive presence of 2-undecenal and 3-hexenal,(Z)- in the early season and the exclusive presence of 2-hexenoic acid and decanal in the late season. The results of PCA showed that there was a distinct separation between S2 and S3 treatments and CK in the early season while S1 and S2 treatments were distinctly separated from CK in the late season. There were 12 and 4 differential flavor volatiles found in the early and late cropping seasons, respectively. Compared with CK, S2 treatment significantly increased the content of 10 flavor volatiles including 2-acetyl-1-pyrroline, benzaldehyde, 2-hexenoic acid, hexanoic acid, octanal, 2-octenal, (E)-, heptanal, 2,4-heptadienal,(E,E)-, 3-hexenoic acid,(E)-, and n-hexadecanoic acid. In addition, the effects of SeNP on flavor volatiles varied between different cropping seasons, which indicated that climate had a great impact on the formation of flavor volatiles in aromatic rice plants and thus, more studies should be conducted to investigate the effects of climate changes on rice aroma as well as related strategies in the future.

## Figures and Tables

**Table 1:** The climate conditions of the experimental site during the field experiment.

Cropping Season	Month	Mean Temperature (°C)	Mean Maximum Temperature (°C)	Mean Minimum Temperature (°C)	Precipitation (mm)
Early season					
	April	23.63	28.19	18.76	77.72
	May	27.39	31.82	22.27	221.49
	June	29.32	34.46	25.16	354.33
Late season					
	August	29.82	34.83	25.02	237.49
	September	28.97	33.41	24.77	427.99
	October	25.43	30.14	20.36	62.99

**Table 2:** The information for flavor volatiles detected in aromatic rice according to the worldwide database of flavor molecules (https://cosylab.iiitd.edu.in/flavordb2/ (accessed on 26 November 2024), Fla-vorDB2).

Cropping Seasons	Common Name	PubChem ID	Flavor Profile	Molecular Formula
Early season				
	Hexanal	6184	leafy, grass, sweaty, tallow, fat, fresh, fruit, aldehydic, green	C6H12O
	3-Hexenal, (Z)-	643941	apple, fat, fruit, leaf, grassy, weedy, green	C6H10O
	Butanoic acid, 2-methyl-	8314	sour, sweat, acid, strawberry, roquefort cheese, pungent, cheese	C5H10O2
	Hexanoic Acid	8892	fat, sour, sweat, cheese	C6H12O2
	O-Xylene	7237	geranium	C8H10
	p-Xylene	7809	sweet	C8H10
	Heptanal	8130	citrus, ozone, fat, herbal, fresh, wine-lee, rancid, aldehydic, green	C7H14O
	2-Acetyl-1-Pyrroline	522834	roast, nut, roasted, ham, sweet, nutty	C6H9NO
	Benzaldehyde	240	cherry, almond, sweet, burnt sugar, sharp, strong, bitter	C7H6O
	1-Octen-3-Ol	18827	raw, fishy, oily, earthy, fungal, chicken, mushroom, green	C8H16O
	Octanal	454	lemon, citrus, soap, orange peel, fat, waxy, aldehydic, green	C8H16O
	2,4-Heptadienal,(E,E)-	5283321	hazelnut, cinnamon	C7H10O
	2-Hexenoic acid	5282707	fat, Must	C6H10O2
	D-Limonene	440917	mint, lemon, citrus, orange, fresh, sweet	C10H16
	2-Octenal, (E)-	5283324	nut, fat, herbal, fresh, green, banana, waxy, leaf, cucumber	C8H14O
	4-Nonenal, (E)-	5283337	fruit	C9H16O
	Nonanal	31289	citrus, lime, orange peel, rose, fat, green, fishy, waxy, fresh, peely, aldehydic, orris, grapefruit	C9H18O
	2-Nonenal, (E)-	5283335	waxy, green, fat, paper, melon, cucumber	C9H16O
	Dodecane	8182	alkane	C12H26
	Decanal	8175	citrus, soap, orange peel, tallow, waxy, floral, sweet, aldehydic	C10H20O
	2-decenal,(E)-	5283345	orange, coriander, rose, tallow, waxy, oily, fat, earthy, floral, aldehydic, mushroom, green	C10H18O
	2-Undecenal	5283356	citrus, soap, orange peel, fat, fresh, sweet, fruit, green	C11H20O
	Tetradecane	12389	alkane, waxy, mild	C14H30
	Vanillin	1183	vanilla, chocolate, sweet, creamy	C8H8O3
	n-Hexadecanoic acid	985	fat, slightly waxy	C16H32O2
Late season				
	Hexanal	6184	leafy, grass, sweaty, tallow, fat, fresh, fruit, aldehydic, green	C6H12O
	2-Butanone	6569	ethereal, ether, fruit, acetone, camphor	C4H8O
	2-Hexenal, (E)-	5281168	leafy, apple, cheesy, vegetable, fat, banana, rancid, sweet, plum, fruit, aldehydic, almond, green	C6H10O
	Ethylbenzene	7500	sweet	C8H10
	o-Xylene	7237	geranium	C8H10
	Heptanal	8130	citrus, ozone, fat, herbal, fresh, wine-lee, rancid, aldehydic, green	C7H14O
	2-Acetyl-1-pyrroline	522834	roast, nut, roasted, ham, sweet, nutty	C6H9NO
	Benzaldehyde	240	cherry, almond, sweet, burnt sugar, sharp, strong, bitter	C7H6O
	1-Octen-3-Ol	18827	raw, fishy, oily, earthy, fungal, chicken, mushroom, green	C8H16O
	Hexanoic acid	8892	fat, sour, sweat, cheese	C6H12O2
	Octanal	454	lemon, citrus, soap, orange peel, fat, waxy, fat, aldehydic, green	C8H16O
	2-Hexenoic acid	5282707	fat, must	C6H10O2
	D-Limonene	440917	mint, lemon, citrus, orange, fresh, sweet	C10H16
	2-Octenal, (E)-	5283324	nut, fat, herbal, fresh, green, banana, waxy, leaf, cucumber	C8H14O
	Nonanal	31289	citrus, lime, orange peel, rose, fat, green, fishy, waxy, fresh, peely, aldehydic, orris, grapefruit	C9H18O
	Acetophenone	7410	mimosa, hawthorn, sweet, acacia, almond, pungent, hawthorne, chemical, flower, bitter, must	C8H8O
	4-Nonenal, (E)-	5283337	fruit	C9H16O
	2-decenal,(E)-	5283345	orange, coriander, rose, tallow, waxy, oily, fat, earthy, floral, aldehydic, mushroom, green	C10H18O
	Indole	798	burnt, jasmine, mothball, animal, fishy, floral, moth ball, naphthelene, honey, fecal	C8H7N
	2-Undecenal	5283356	citrus, soap, orange peel, fat, fresh, sweet, fruit, green	C11H20O
	Vanillin	1183	vanilla, chocolate, sweet, creamy	C8H8O3
	n-Hexadecanoic acid	985	fat, slightly waxy	C16H32O2

## Data Availability

Data is contained within the article (The original contributions presented in the study are included in the article, further inquiries can be directed at the corresponding author).
